# Optical Properties, Synthesis, and Potential Applications of Cu-Based Ternary or Quaternary Anisotropic Quantum Dots, Polytypic Nanocrystals, and Core/Shell Heterostructures

**DOI:** 10.3390/nano9010085

**Published:** 2019-01-10

**Authors:** Xue Bai, Finn Purcell-Milton, Yuri K. Gun’ko

**Affiliations:** School of Chemistry and CRANN Institute, Trinity College Dublin, Dublin 2, Dublin, Ireland; baix@tcd.ie (X.B.); fpurcell@tcd.ie (F.P.-M.)

**Keywords:** quantum dots, polytypic nanocrystals, core/shell heterostructures, luminescent solar concentrators, bioimaging, light emitting diodes

## Abstract

This review summaries the optical properties, recent progress in synthesis, and a range of applications of luminescent Cu-based ternary or quaternary quantum dots (QDs). We first present the unique optical properties of the Cu-based multicomponent QDs, regarding their emission mechanism, high photoluminescent quantum yields (PLQYs), size-dependent bandgap, composition-dependent bandgap, broad emission range, large Stokes’ shift, and long photoluminescent (PL) lifetimes. Huge progress has taken place in this area over the past years, via detailed experimenting and modelling, giving a much more complete understanding of these nanomaterials and enabling the means to control and therefore take full advantage of their important properties. We then fully explore the techniques to prepare the various types of Cu-based ternary or quaternary QDs (including anisotropic nanocrystals (NCs), polytypic NCs, and spherical, nanorod and tetrapod core/shell heterostructures) are introduced in subsequent sections. To date, various strategies have been employed to understand and control the QDs distinct and new morphologies, with the recent development of Cu-based nanorod and tetrapod structure synthesis highlighted. Next, we summarize a series of applications of these luminescent Cu-based anisotropic and core/shell heterostructures, covering luminescent solar concentrators (LSCs), bioimaging and light emitting diodes (LEDs). Finally, we provide perspectives on the overall current status, challenges, and future directions in this field. The confluence of advances in the synthesis, properties, and applications of these Cu-based QDs presents an important opportunity to a wide-range of fields and this piece gives the reader the knowledge to grasp these exciting developments.

## 1. Introduction

Quantum dots (QDs) are very important light emitting nanomaterials, which have been intensively studied for several decades. Several excellent reviews have covered the theoretical and experimental investigations of the synthesis, optical properties, and multimodal applications in the past several years in great detail [1,2,3,4]. The most well-explored systems are the II-VI type Cd-based QDs [5,6], and corresponding Cd-based heterostructures [7,8,9]. More recently, Cu-based multicomponent (ternary, quaternary, and quasi-quaternary) QDs have become an area of great attention due to their specific advantages relative to its Cd-based counterparts and a range of other unique and important properties. Firstly, Cu-based multicomponent QDs show even greater absorption and emission tunability comparing to Cd based systems since they not only display a size dependent bandgap, but also display the property of off-stoichiometry, meaning the optical properties of Cu-based ternary or quaternary QDs can be tuned via varying of the ratio of cations that compose the QD. Secondly, the absence of toxic heavy metals (Cd or Pb) in Cu-based ternary or quaternary QDs shows particular advantages due to reduced environmental impact and better suitability for biological applications. Thirdly, Cu-based multicomponent QDs possess enhanced stability against the external environment (oxygen, moisture, and photo-irradiation), especially after inorganic shell coating with large band gap semiconductors, such as ZnS [10,11,12]. Thus, Cu-based multicomponent QDs are very promising candidates to replace Cd-based QDs.

It is well known that the optical properties of QDs are not only size and composition dependent, but are also strongly determined by the resulting shape, with several morphologies demonstrating this, including nanorods (NRs), nanoplatelets (NPLs), and tetrapods (TPs). Their synthesis is well explored for Cd-based systems [7,13,14,15,16,17,18], with these nanostructures, especially NRs and TPs, showing increased electrical conductivity compared to core, quasi-spherical nanoparticles, and therefore are very appealing for applications requiring efficient charge transfer [19]. CdSe/CdS dot in rods, rod in rods, and CdSe nanoplatelets have been shown to demonstrate polarized emission for use in a liquid crystal display, acting as optical funnels, absorbing across a significant range of unpolarised wavelengths and then emitting strongly linearly polarised emission at a specific wavelength depending on the specific nanostructure [20]. Semiconductor nanorods may also serve as an excellent gain media for amplified spontaneous emission (ASE) and polarized lasing, as was demonstrated for CdSe/ZnS core/shell rods [21]. For CdSe/CdS seeded nanorods, a significant decrease in the Auger recombination rate is expected, which make these structures favourable for such applications [22]. It has also been demonstrated that heterostructures function very effectively, showing a structurally related larger Stokes’ shift while maintaining high quantum yields. In addition, a number of heterostructured cadmium-based NPs, when combined *in situ* with tips of noble metals, demonstrate huge potential in the field of solar to fuel conversion [23].

Rapid development of techniques to synthesize anisotropic quantum nanostructures have recently enabled the preparation of Cu-based multicomponent NRs, NPLs, and TPs structures. According to the number of phases that exists in these Cu-based multicomponent nanostructures, they can be divided into two categories. The first category is represented by a pure single crystal phase, termed as Cu-based ternary or quaternary anisotropic nanostructure. The second category is heterostructures, formed by at least two phases, which exist as a nanocrystal, including polytypic QDs and core/shell heterostructures. Polytypic QDs refer to QDs that are prepared through the controlled engineering of different polymorphs in one single nanostructure. As for heterostructures, they are prepared via coating of a core semiconductor with a shell of an alternative semiconductor.

To the best of our knowledge, to date, there is no comprehensive review that summarises the development of these systems, including unique optical properties, the range of morphologies produced, techniques for their synthesis, and potential applications of luminescent Cu-based multicomponent anisotropic and heterostructured nanocrystals. Therefore, the major goal of this review is to cover this field in depth, including: preparation of Cu-based multicomponent anisotropic core QDs, heterostructures and polytypic NCs, their optical properties, and their potential applications in areas, such as luminescent solar concentrators, LEDs, and bio-imaging.

In this review, we cover the following Cu-based ternary and quaternary semiconductors that we judge the most relevant and therefore important, with a summary of properties of some selected ternary and quaternary metal chalcogenide materials shown in Table 1 [24]. Ternary Cu-In-Se (CISe) and Cu-In-S (CIS) compounds are direct semiconductors with relatively narrow band gaps (1.05 and 1.53 eV, respectively) and high absorption coefficients (10^5^ cm^−1^), making them promising absorbing materials in solar cells. In bulk, they exhibit the chalcopyrite (CH) crystal phase at room temperature. In addition, the zinc blende (ZB) and wurtzite (WZ) crystal structures are also present, but only stable at high temperatures. To date, CISe and CIS QDs with CH [25,26,27,28,29,30,31], WZ [32,33,34,35,36,37,38] and ZB [39,40,41,42,43] crystal structures have been prepared, all of which appear stable and are produced through variations of the reaction conditions. Another important class involves Cu-Sn-Se (CTSe) and Cu-Sn-S (CTS) compounds, which are direct semiconductors and show a tendency to crystalize in a large range of phases and structural forms, such as cubic sphalerite-like phase, an orthorhombic structure, and also a metastable WZ phase [44]. In comparison, CTSe QDs with WZ [45] and ZB [46] crystal structures have been prepared, while CTS QDs with ZB [47], WZ [48], and kesterite (KS) [49] crystal structures are available.

Considering quaternary systems, CuIn_1−x_Ga_x_S (CIGS), Cu_2_ZnSnS_4_ (CZTS), and Cu_2_ZnSnSe_4_ (CZTSe) are the most widely studied of quaternary semiconductors because they are made of earth abundant and non-toxic elements and their direct band gaps cover the optimal energy range of photovoltaic applications. In bulk, the most stable structure of CIGS compounds is the CH structure [50], while CZTS and CZTSe adopt the tetragonal KS structure. In comparison, CZTS QDs have been produced with the KS [51,52,53], WZ [54,55,56,57], and ZB [58,59] crystal structures and CZTSe QDs have been prepared with the stannite (ST) crystal structure [60,61,62] and CIGS QDs have been synthesised in the CH [63,64] and WZ [50,65] phases. The introduction of zinc species in ternary CISe and CIS systems is straightforward and leads to Cu-In-Zn-Se (CIZSe) and Cu-In-Zn-S (CIZS) quaternary systems with a tuneable band gap and high photoluminescence quantum yields (PLQYs) [66,67,68]. CIZSe QDs with CH crystal structure have been prepared [69,70] and CIZS QDs with CH [71,72,73,74] and ZB [67] structures have also been reported. Therefore, in our review, we would like to consider ternary Cu-III-VI and Cu-IV-VI semiconductors and quaternary Cu-II-IV-VI and Cu-III-III-VI semiconductor nanostructures. Here, we discuss unique optical properties (e.g., high photoluminescent quantum yields, size-dependent bandgap, composition-dependent bandgap, broad emission range, large Stokes’ shift, and long photoluminescent (PL) lifetimes) of the Cu-based multicomponent QD nanostructures, their synthesis (including various strategies to control their shapes and morphologies), and their applications, such as luminescent solar concentrators (LSCs), bioimaging, and light emitting diodes (LEDs). Finally, we would like to provide perspectives on the overall current status, challenges, and future directions in this field.

## 2. Unique Optical Properties of Cu-Based Multicomponent QDs

### 2.1. Emission Mechanism

**Figure 1:**
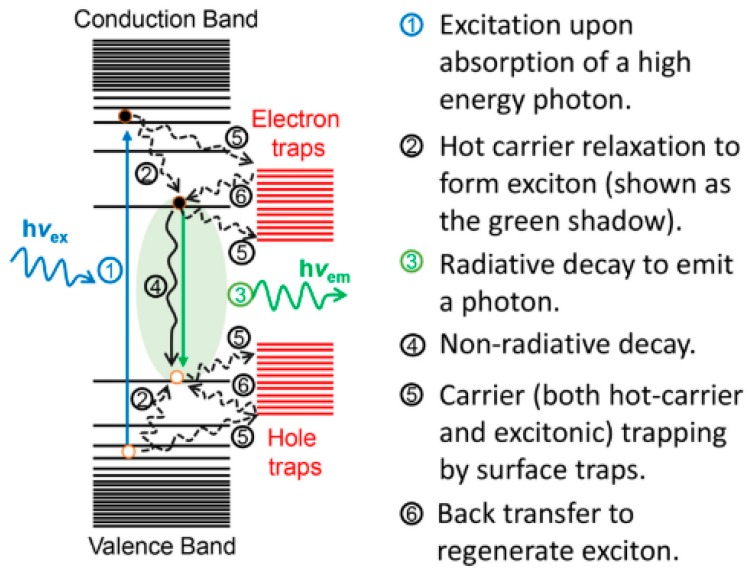
Scheme of the basic processes involving various excited states in a quantum dot upon photoexcitation. Reproduced with permission from [75]. Copyright American Chemical Society, 2017.

The major fundamental photophysical processes taking place in QDs are schematically presented in Figure 1. Upon photon excitation, an electron can be promoted to a certain energy level in the conduction band and is termed as a hot carrier that leaves a hole in the valence band at the same time. Following this, there are two main pathways for the hot carriers to release energy. The first way is the relaxation of the electron-hole pair to the band edge position to form excitons, the other possibility is trapping of the hot carriers by the surface defects. The formed excitons also have two potential channels to release the energy; one is releasing the energy by radiative decay resulting in an emission, and the other way is the non-radiative decay, normally resulting in the thermalisation and heat release. The trapped by the surface traps hot carriers can also back transfer to regenerate excitons that can be followed by radiative or non-radiative electron-hole recombinations.

For Cu-based ternary or quaternary QDs, the emission mechanism is still under debate. There are different photoluminescence mechanisms that have been proposed due to the complexity of the different crystal structures and also the large range of non-stoichiometric compositions. Among them, there are two widely accepted possible luminescence mechanisms; one is donor-acceptor pair (DAP) recombination, and the second one is free-to-bound recombination.

The DAP recombination was established in the early 1950s [76,77]. It involves a deep acceptor level and a relatively shallow donor level. Among ternary Cu chalcogenide systems, CIS is the most investigated compound. It was suggested by Castro et al. [78], Chen et al. [79], and Kraatz et al. [80] that the presence of a donor−acceptor pair was a plausible reason, due to their faster relaxation pathways, as compared to band edge recombination. DAP recombination involved a deep acceptor level identified as either a copper vacancy (V_Cu_, formed under In^3+^-rich growth conditions) or V_In_ or Cu_In_ defects (formed under Cu^+^-rich growth conditions), and a relatively shallow donor level identified as In_Cu_, V_S_, or an interstitial In (In_i_) [81,82,83,84].

When describing free-to-bound recombination, one of the carriers is delocalized in the conduction band or valence band and the other carrier is localized at a defect [85,86,87]. For CIS, the electron is in the conduction band and the hole is localized [88,89,90]. It also has been proposed that the DAP and free-to-bound mechanism coexist as a competitive recombination pathway [85,91,92], with the dominant mechanism determined by the NC stoichiometry and whether the NC is shelled.

### 2.2. Size-Dependent Band-Gap 

According to the Brus equation [93]:

(1) $$\Delta E\left(R\right)=E_{g}\;(R)+\frac{h^{2}}{8R^{2}}\left(\frac{1}{{{m\;}_{e}}^{*}}+\frac{1}{{{m\;}_{h}}^{*}}\right)-1.8e^{2}/4\pi \varepsilon_{0}\varepsilon_{\alpha }R$$

where ΔE is the new band gap energy due to quantum confinement, $E_{g}$ is the bulk band gap energy, R is the radius of the particle, ${m_{e}}^{*}$ is the electron effective mass and ${m_{h}}^{*}$ is the hole effective mass, $\varepsilon_{0}$ is the permittivity of a vacuum, $\varepsilon_{\alpha }$ is the high frequency dielectric constant, e is the charge of the electron.

**Figure 2:**
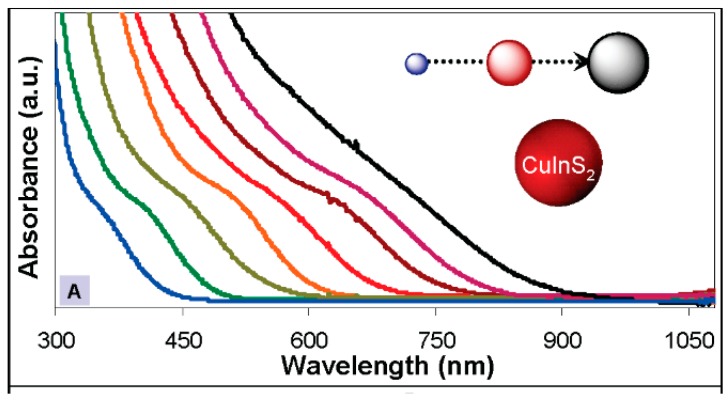
The emission of copper indium sulfide (CIS) QDs with different diameters obtained under different reaction temperatures. Reproduced with permission from [94]. Copyright American Chemical Society, 2009. Composition-dependent band-gap.

QDs show size-dependent band gap energy with smaller QDs showing increased band gap energy. The Bohr radius of CIS is 4.1 nm; thus, quantum confinement effects can be observed in CIS NCs up to a size of approximately 8 nm. By changing the sizes of CIS QDs, their absorption and emission can be tuned from visible up to the near infrared region [94] (Figure 2). An excellent example of this size dependent behaviour has been demonstrated using CIZS by Zhang and Xie, who synthesized CIZS QDs with a hot injection method [67]. They used different reaction temperatures (180, 210, and 240 °C) to obtain QDs with a range of different diameters, with the corresponding emission of the sample tuned from 620 to 750 nm by increasing the size of the NCs from 2.0 to 7.0 nm, even though no well-defined exciton absorption peaks were observed for all the sample. In addition, this behaviour has also been demonstrated via variation of the reaction time [95]. Sun et al. observed that the resulting bandgaps of CIGS QDs moved from 1.83 to 1.64 eV when the sizes of the particles increased from 2.9 to 4.3 nm [63]. In addition, the same trends were observed for CZTS QDs [96] and CZTSe QDs [61].

**Figure 3:**
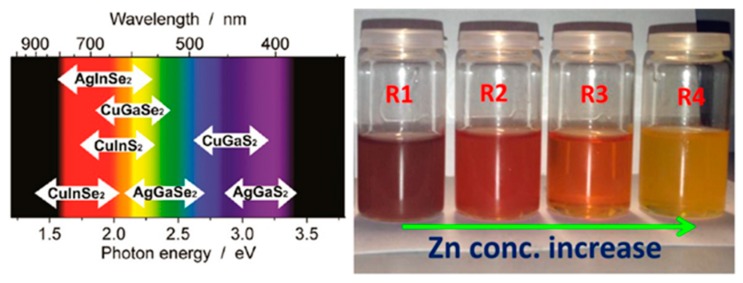
(**Left**) Optical bandgap for I-III-VI type nanocrystals (NCs) in the size range between 2 and 5 nm. Reproduced with permission from [106]. Copyright AIP Publishing, 2009. (**Right**) Photographs of Cu-In-Zn-S (CIZS) nanorods (NRs) with various zinc concentrations. Reproduced with permission from [19]. Copyright American Chemical Society, 2015.

According to Zunger’s theoretical model on defects [97], compared to binary Cd- and Pb-based QDs, Cu-based QDs can bear large stoichiometric tunability. In this way, changing the composition is another effective way to tune the emission of the NCs in addition to varying the size of the sample. This can be done by changing the ratio of the precursors used in certain reactions. Generally, the incorporation of Zn into Cu-based ternary systems causes a blue shift in the absorption and emission spectra. The emission peak of the as-prepared highly luminescent CIZS/ZnS showed great contrast to that of CIS/ZnS NCs [79] and has demonstrated that the emission would blue shift with more Zn incorporated in the Cu-based multicomponent NCs [98,99,100]. In addition, this composition tunability has been demonstrated in heterostructured QDs also by Sing et al. having tuned the composition and therefore band gap of CIZS NRs. Therefore, these CIZS NRs show blue shifting in absorption onset due to an increasing Zn concentration as shown in Figure 3 [19]. Song et al. has demonstrated that the band gaps of the quaternary CIGS QDs systematically increased with a higher Ga concentration between 2.15 (CIS QDs) and 2.60 eV (CGS QDs) [101]. The same trend was also found by Kim et al. and Dilena [65,102]. The photoluminescence spectra of CIZSe QDs display a slight blue-shift with the decrease of the Cu/In ratio with the absorption onset of this sample also exhibiting the same trend as the Cu/In ratio decreased. It is believed that decreasing the Cu concentration in the CIZSe QDs leads to the increase of the band gap [70] and is consistent with the observations from other reports [79,103,104]. The same type of behaviour has also been demonstrated in CZTS QDs, with the bandgap also able to be tuned from 1.56 to 1.83 eV by varying the compositional ratios of Cu and Zn [105].

### 2.3. Broad Emission Range

For binary Cd- or Pb-based QDs, if the size distribution is narrow, the emission range can be characterized with a small HWHM (half width at half maximum). This is more complicated for Cu-based ternary or quaternary QDs, since the chemical composition of one QD can differ from that of another one. Many studies have been carried out to explore the reasons for the broad emission of Cu-based QDs with the PL bandwidths having been investigated by optical microscopic studies of Cu-doped (Cu^+^:CdSe) QDs [107]. Brovelli et al. have also carried out luminescence line narrowing in ensembles of copper-doped ZnSe/CdSe core/shell NCs [108]. In addition, size-selective precipitation has been carried out on CIS NCs, with the aim to narrow the size distribution, while results still showed the overall shape of the UV-Vis absorption and photoluminescence did not change [78]. All the experimental results show that copper-based emission in these materials is intrinsically broad and therefore it is very challenging to achieve small HWHM, for the Cu-based ternary or quaternary multicomponent core/shell structures even when the size distribution is narrow.

There are several reasons for this broad emission. The first is that it is hard to balance the reactivity of the related metal elements in the reaction, which easily results in compositional difference among different particles from the same batch, and the compositional differences give rise to a distribution of the donor-acceptor distances, which corresponds to a broad emission range [108]. The second cause is dependent upon the strong electron-photon coupling, which leads to a large nuclear distortion around the copper in the luminescent excited state [107]. Differences in local environments of Cu-based QDs, especially at the core/shell interface, have also been reported to relate to broad PL emission [108].

### 2.4. Large Stokes’ Shift

The Stokes’ shift is commonly referred to as the difference in energy between the position of the band maxima of the absorption and emission spectra of the same electronic transition and is attributed to the energy difference between the first exciton absorption peak and the emission peak of QDs. In general, Cd-based QDs demonstrate small Stockes’ shifts (0.1–0.2 eV). In contrast, multicomponent copper chalcogenide NCs exhibit large Stokes’ shifts (0.2–0.6 eV). A theoretical study of the band structure of small CH CIS NCs based on the multiband effective-mass approximation was developed by A. Shabaev [109]. It was concluded that it was the intrinsic optical property for CIS NCs to have a significant Stokes shift. Li et al. suggested that the large Stokes’ shift between the PL band and the band-edge absorption feature was not in accordance with either band-edge recombination or recombination between two localized states; instead, it involved a transition from a conduction-band state to an intra band state [88], which is consistent with the free-to-bound emission mechanism. However, as is suggested by Castro et al., the distinct energy shift between the excitation and emission peaks is consistent with the DAP recombination mechanism [78].

No matter the DAP or free-to-bound mechanism, the typical broad emission is related to the various types of intrinsic defects (lattice vacancies or other point defects) that are present in Cu chalcogenide systems, which not only leads to possibilities of compositional inhomogeneities within QDs, but also consolidates many kinds of defect-based, nonradiative decay pathways in their luminescence.

For type I Cu-based core/shell structures, it is important to produce the large Stokes’ shift, since the shell with a larger bandgap can dominate the absorption spectrum, while the emission originates from the core with a narrower bandgap, therefore, the energy difference gives rise to a large Stokes’ shift, via band gap engineering. Doped Cu-based QDs also possess a large Stokes’ shift due to the intra-band energy level introduced by this dopant.

### 2.5. Long PL Lifetime

Cu-based ternary or quaternary, core/shell heterostructures possess surprisingly long-lived emission, in the order of magnitude of several hundred nanoseconds, and it is believed that the relatively long decay time is typical for defect related emission of Cu-based systems [110]. Li and Pandey prepared CIS-based core/shell structures [88] and a very long PL lifetime (ca. 500 ns) was observed for the core/shell NCs. The authors attributed the long-lived emission to a large disparity in the localization volumes of the electron and the hole, which would lead to a slowed radiative decay, though further study needs to be done to elucidate this question more clearly. This increased lifetime is suitable for some applications, especially for bioimaging, since when using Cu-based NCs, long PL lifetimes help reduce the background signals from autofluorescent biological tissue, via time gating approaches [111].

## 3. Synthesis of Cu-Based Ternary or Quaternary Anisotropic Nanostructures

### 3.1. Preparation of Nanorods

**Figure 4:**
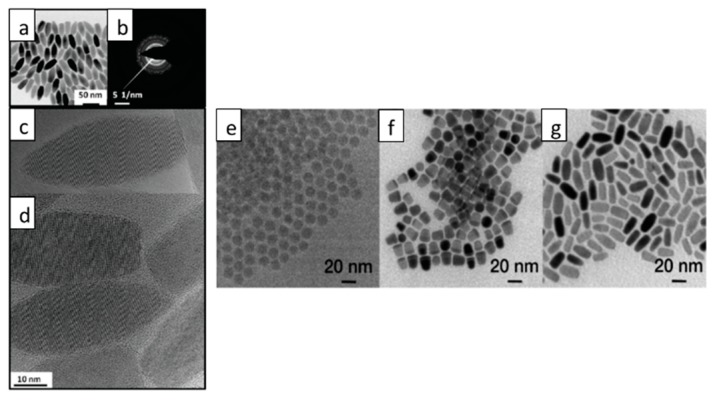
Transmission Electron Microscope (TEM) images of typical Cu-based ternary nanorod structures, (**a**–**d**) TEM images of CIS NRs with the hot-injection method. Reproduced with permission from [112]. Copyright American Chemical Society, 2010. (**e**–**g**) TEM images of CIS NRs with the hot-injection method. Reproduced with permission from [113]. Copyright American Chemical Society, 2014.

NRs are nanostructures with one-dimensional geometry and this is one of the simplest heterostructures reported in literature for a range of Cu-based ternary and quaternary structures, which have been produced using the well-known hot-injection and heating-up methods. TEM images of Cu-based ternary and quaternary NRs structures are shown in Figure 4. Though similar in structure and morphology, much variance in the exact mechanism of production is a topical subject in the literature today.

In 2010, Kruszynska and Borchert produced the first CIS NR structure via a hot-injection method [112]. These NRs were uniform in size and shape, having a mean width of 19.1 ± 1.4 nm and a length of 44.8 ± 3.8 nm. HRTEM images of single CIS NRs showed that WZ CIS structures were obtained. The authors have also interpreted the growth mechanism for the CIS NR structure, by the initial formation of Cu_2_S nanoparticles, which served as seeds for anisotropic growth of CIS NRs. Li et al. synthesized CIS NRs with an alternative hot-injection method [113]. They proposed that the growth of CIS NRs started with the formation of Cu_2_S seeds; intriguingly, two different growth mechanisms of the CIS NRs were observed in their case, dependent upon the size of the Cu_2_S seeds in this work (Figure 4), with the size of the formed Cu_2_S seeds controlled via the amount of oleic acid used in the reaction. It was found that smaller seeds (4 nm) were gradually converted to CIS NRs with the incorporation of indium, while in contrast, larger seeds (8 nm) served as the plate for the nucleation of CIS, resulting in a Cu_2_S-CIS hybrid nanostructure as intermediates, followed by the two-part fusing into CIS NRs.

**Figure 5:**
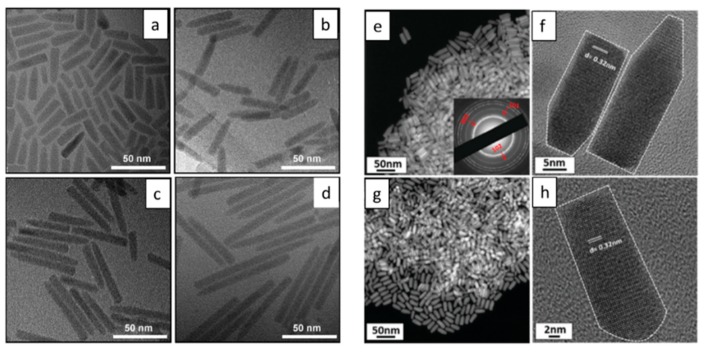
TEM images of Cu-based quaternary NRs, (**a**–**d**) CIS-ZnS alloyed NRs. Reproduced with permission from [114]. Copyright WILEY-VCH Verlag GmbH & Co. KGaA, Weinheim, 2012. (**e**–**h**) CZTS NRs with the hot-injection method. Reproduced with permission from [116]. Copyright American Chemical Society, 2012.

Some efforts have been focused on the synthesis of Cu-based quaternary NRs. The synthesis of alloyed (ZnS)_x_(CuInS_2_)_1−x_ semiconductor NRs was reported by Ye and Regulacio [114] (Figure 5). The obtained NRs adopt a hexagonal WZ crystal structure and their formation involved the initial nucleation of hexagonal Cu_2_S. Alloyed WZ CIZS NRs were prepared by Li et al. with a similar heating-up method [115]. In their work, the growth of the NRs started from the formation of Cu_2_S particles, which served as seeds for the further generation of CIZS with an elongated shape. The diameter of the NRs did not change during the growth process, while their length increased. Upon changing the zinc concentration, the diameter of the NRs could be tuned from 5.9 to 7.2 nm, while the length could be varied from 13.3 to 21.4 nm. In addition, synthesis of WZ CIZS NRs with a hot-injection method was also reported by Singh and Coughaln [19]. The NRs demonstrated a substantial increase in the length from 30 to 120 nm, while the diameter remained at 8–9.58 nm when the zinc concentration in the reaction mixture increased. The obtained NRs could be assembled in both lateral and perpendicular arrays, which may be of significant interest for photocatalysis, photovoltaics, and photoemissive applications. There are several reports about the synthesis of CZTS NRs [116,117], and even Mn^2+^, Co^2+^, and Ni^2+^ doped CZTS NRs [118]. All of these obtained CZTS NRs possess a metastable WZ crystal structure. Their formation began with the formation of spherical of Cu_2_S particles, and as the reaction proceeded, the anisotropic NR shape evolved with the incorporation of other metal ions. The influence of reactivity between Zn and S on the crystal structure of obtained NRs was explored by Zou and Su in the synthesis of CZTS NRs [119]. In their work, a highly reactive sulfur precursor and metal acetates were used to prepare WZ CZTS NRs. On the other hand, using a low-reactivity sulfur precursor or metal chlorides, CZTS NRs in the KS phase were produced.

Singh et al. produced WZ CIGS NRs via a hot-injection method and the obtained CIGS NRs were assembled into 2D or 3D superstructures [120]. The prepared CIGS NRs were highly monodispersed, measuring 11 ± 0.5 nm in width and 24 ± 1 nm in length. The CIGS NR assemblies could be extended over device scale areas with high degrees of order, which is attractive for applications, such as photoabsorbers. Later, the same group checked the synergistic role of the dopants (Sb^3+^) on the morphology of CIGS NRs [121]. It was found that the presence of Sb^3+^ as a dopant in the reaction solution for CIGS NRs caused end-to-end fusion of NR pairs into nanodumbbells at high yield. Without Sb^3+^ in the reaction solution, CIGS with normal rod shapes were obtained. It turned out that the dopant (Sb^3+^) catalysed the incorporation of gallium, tuning the reaction kinetics, and leading to the formation of the specific CIGS nanodumbbells.

Overall, the formation of binary Cu_2_S particles is a key initial step for the synthesis of ternary or quaternary NRs. These Cu_2_S particles serve as a template for the anisotropic development with the incorporation of other metal ions into the structure to produce the final NR structure.

### 3.2. Synthesis of Nanoplatelets

Nanoplatelets (NPLs) are an important category in Cu-based multicomponent anisotropic nanostructures, because NPLs can easily form stable inks for a range of applications [122]. The preparation of NPLs further demonstrate the possibility of shape and size control in Cu-based multicomponent NCs, with Cu-based ternary (CISe [122,123,124,125], CIS [42,126,127,128,129], CTSe [49]), and even quaternary (CZTS [130]) NPLs having been produced in recent years. A range of techniques are employed within these works, including hot-injection, template-based, and the emerging approach of cation-exchange. The technique of cation exchange has become an effective tool to synthesize a range of structures and has shown particular success in the synthesis of Cu-based ternary or quaternary NPLs, giving access to NPLs that cannot be obtained via normal solution synthesis methods (heating-up and hot-injection), such as hollow nanostructures [131,132,133].

**Figure 6:**
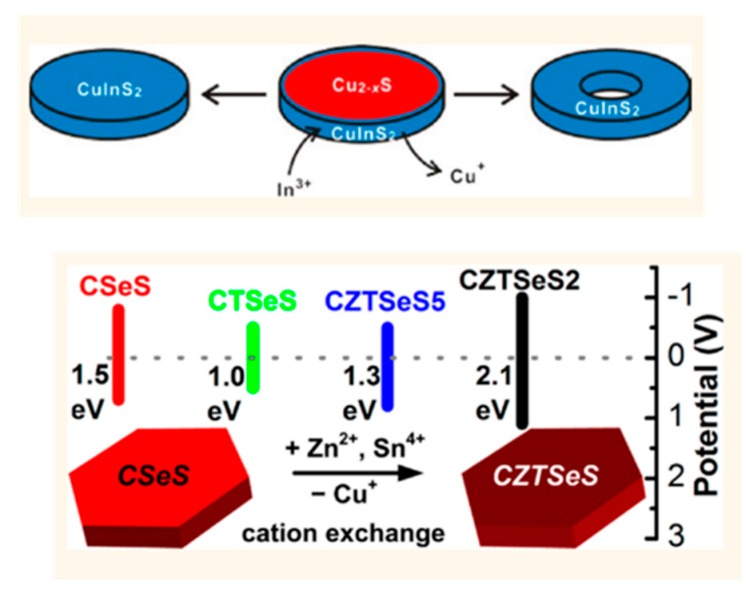

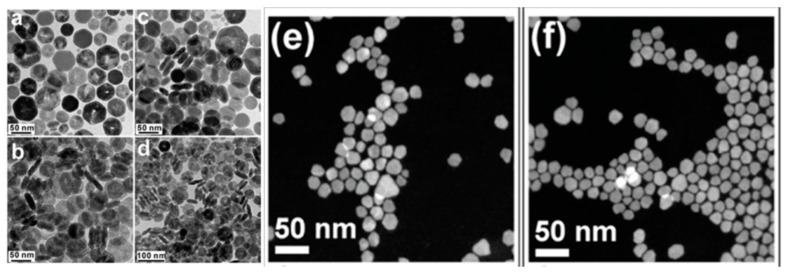
Top: Scheme of the synthesis of copper indium sulphide (CIS) [129] and copper zinc tin selenide–sulfide (CZTSeS) [130] nanoplatelets (NPLs) via cation exchange technique. Bottom: (**a**–**d**) TEM images of CIS nanocrystals. and (**e**–**f**) scanning TEM images of CZTSeS NPLs via cation exchange technique. Reproduced with permission from [129]. Copyright American Chemical Society, 2015. Reproduced with permission from [130]. Copyright American Chemical Society, 2014.

Mu and Wang prepared hollow CIS NPLs via cation exchange, since the diffusion rate of Cu^+^ out of the structure is faster than the incorporation rate of In^3+^ with certain ligands in the reaction with the well-known “Kirkendall effect” thus observable [129]. Cu-Zn-Sn-Se-S (CZTSeS) NPs were also prepared via a similar partial cation exchange reaction [130] (Figure 6). Recently, Liu et al. used covellite Cu_2−x_S as the template to produce a range of ternary NPs, including CIS, Cu-Fe-S (CFS), Cu-Ga-S (CGS), Cu-Ge-S (CGeS), and CTS NPLs, in addition, quaternary CIZS and CZTS NPLs [134] were also produced. From this work, they concluded that trivalent and tetravalent cations (In^3+^, Ga^3+^, Fe^3+^, Sn^3+^, and Ge^4+^) could be incorporated into covellite Cu_2−x_S to form ternary alloy NPLs, while in contrast, the incorporation of divalent cations (Zn^2+^, Cd^2+^, and Pb^2+^) would cause partial or complete cation exchange to produce heterogeneous or Cu-free NCs.

## 4. Synthesis of Cu-Based Multicomponent Polytypic QDs

Polytypic NCs can be obtained through the controlled engineering of different polymorphs in one single nanostructure, which is also a very efficient technique to produce a range of anisotropic QDs. The key point of a general strategy of the approaches to prepare polytypic QDs is that one crystal phase forms first under certain reaction conditions followed by changing the conditions to grow an alternative crystal structure. With the development of colloidal synthesis techniques, polytypic NCs can now be achieved in a range of Cu-based ternary and quaternary NR and TP nanostructures.

**Figure 7:**
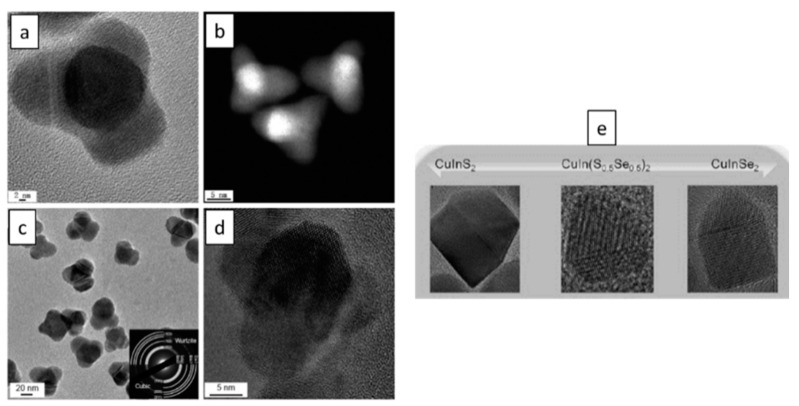
TEM images of Cu-based ternary polytypic quantum dots (**a**–**d**) Cu_2_SnSe_3_ polytypic tetrapods. Reproduced with permission from [136]. Copyright American Chemical Society, 2013. (**e**) CIS, Cu-In-Se-S (CISeS), and CISe polytypic NRs. Reproduced with permission from [135]. Copyright American Chemical Society, 2016.

Wu and Chen explored the synthesis of polytypic NCs of the Cu-based ternary systems, including polytypic CISe and CIS (Figure 7) [135]. Each obtained polytypic NC consisted of a WZ column and a ZB tip. The growth mechanism was studied for the polytypic CIS, which were prepared by a hot-injection method. First, the Cu_31_S_16_ NCs were nucleated at low temperatures, followed by the diffusion of indium ions into Cu_31_S_16_ particles to form WZ CIS nanocylinders, and then ZB CIS was nucleated on the (001) facet of WZ CIS nanocylinders to form a bullet-shaped polytypic NC. A different method was employed to prepare CISe polytypic NCs, with the cation precursor and anion precursor solutions being prepared in separate flasks, then mixed at 70 °C, with the temperature increased to 280 °C, and kept at that temperature for 30 min to produce the final polytypic structure. It is also interesting to note that the growth mechanism of CISe was different from that of CIS polytypic NCs. For CISe polytypic NCs, WZ CISe nanoparticles formed at relatively lower temperatures, followed by growth into a prism morphology, which was then followed by the nucleation of ZB CISe tips onto the prisms at higher temperatures.

CTSe TP polytypic structure have been reported by Wang (Figure 7) [136] and was produced via a hot-injection technique, with an average arm diameter of 28.2 nm and a length of 17.5 nm having been determined. The length of the TP arms were typically always comparable to the core dimensions regardless of the growth time. The obtained CTSe TPs nucleated with a cubic core with four short WZ arms. The critical element of this synthesis was the initial high temperature injection that favoured nucleation in the ZB crystal structure and subsequent growth at a slightly lower temperature favouring the hexagonal phase. One year later, they managed to produce linear CTSe polytypic NCs by another non-injection method [46]. WZ CTSe particles were formed at lower temperatures, which was then followed by the epitaxial growth of two ZB tips. In another synthesis, CZTSeS polytypic NCs were formed, producing ellipsoid, arrow, and bullet shapes, using a coordinating solvent (oleylamine), trioctylphosphine oxide (TOPO), and phosphonic acids in the reaction [137]. At the same time, instead a non-coordinating solvent (ODE) and ligand (TOPO) were used, and CZTSeS quantum nanostructures in arrow or rod shapes as single NCs were produced. Their work confirmed the possibility to engineer the anisotropic shape and phase of Cu-based ternary or quaternary nanostructures in a controlled manner.

Fan et al. prepared quaternary linearly arranged CZTSSe NCs [138]. This NR structure consisted of two ZB ends and one WZ centre part. At low temperatures (150 °C), Se precursor was injected into the reaction vessel with Cu, Zn, and Sn precursors, and the reaction started with the nucleation of WZ structure. Subsequent epitaxial growth of the ZB structure was facilitated by the increased reaction temperature (280 °C). Finally, the ZB/WZ/ZB linear arranged NCs were obtained. Interestingly, it was found that this NR structure with different phase ratios could be produced by changing the reaction temperature (240–320 °C). The dominant phase of this ZB/WZ/ZB linear arrange structure converted from the WZ phase (240 °C) to ZB phase (320 °C). It is suggested that this can be a new band-gap tuning approach. Quaternary Cu_2_CdSn(S_1−x_Se_x_)_4_ polytypic NCs with WZ cores and ZB arms were made by Wu et al. [139]. The obtained NCs had two morphologies: one portion was rugby ball-like NCs, while the other portion was bullet-like NCs. The authors found that the ratio of the rugby ball-like and bullet-like NCs could be tuned through changing the amount of Cd precursor in the reaction solution. The amount of Cd precursor had great influence on the reactivity difference between (0002) facets and (000-2) facets of the formed WZ cores. It was proposed that the diversity of the two facets increased with the mole ratio of Cd, then the ZB structure would be easier to nucleate at (000-2) facets to give bullet-like polytypic NCs. Even though this work involved heavy metal Cd, the study approach could instruct the future works based on Cu in this area. There are also some other multicomponent polytypic NCs that have been prepared, including the Cu_2_Cd_x_SnSe_y_ polypods [140] and polytypic Cu_2_GeSe_3_ nanostructures [141].

Overall, in these syntheses, the shape evolution of polytypic nanostructures is dictated by independently controlling the respective growth rates of either the ZB or WZ regions in the polytypic system. For some certain reactions, temperature is the key point in the nanostructure engineering process. Sometimes, it is effective to use specific ligands to tune the reactivity of precursors to facilitate or inhibit the formation of either the WZ or ZB crystal structure. In general, for these polytypic syntheses, when the initial core is of the WZ form first, then a NR polytypic NCs can be grown using ZB growth, while if ZB particles form at the very beginning, then polytypic branching NCs can be prepared.

## 5. Synthesis of Cu-Based Multicomponent Heterostructures

Heterostructured NCs can also be produced via the coating of a core semiconductor with a shell of an alternative semiconductor. Using this approach not only the structure can be profoundly modified, but the electronic structure can be finely controlled also via the electronic band alignments of the core and shell semiconductors when coating a semiconductor. Based on the relative bandgaps and positions of the bandgaps of the involved semiconductors (materials that have been chosen to build core/shell structure), the heterostructures can be divided into three types, termed type I, reverse type I, and type II [142]. Type I heterostructures are the most commonly utilised, since this can hugely enhance the PL properties by passivating surface defects, which act as non-radiative pathways, and also by protecting the excitons from the external environment [143]. An important consideration when producing heterostructures is related to difference in the lattice constants of the core and shell material, which can produce distinct lattice strain at the interface of the core and shell, which increases as the thickness of the shell grows, resulting in crystal defects, and reduced PLQY of the core/shell structures. In addition, if crystal structures are distinctively different, it may prove impossible to produce uniform growth and instead the formation of polytypic crystals takes place, with shell material only growing in specific areas upon the original core material [139].

There are three distinct and important types of multicomponent heterostructures that we will discuss: Cu-based multicomponent spherical core/shell heterostructures, dot-in-rod heterostructures, and TP heterostructures.

### 5.1. Synthesis of Cu-Based Spherical Core/Shell Heterostructures

The preparation of Cu-based multicomponent spherical heterostructures is quite a well-developed field regarding the control of the size and composition. ZnS is normally the optimal choice for the shell coating of Cu-based multicomponent QDs, due to the minor lattice difference between them and the production of a type I band alignment.

The typical examples of Cu-based ternary heterostructures are CISe- and CIS-based core/shell structures, because they constitute non-toxic elements, display the bright emission in the near infrared region, have high absorption coefficients, and relatively long photoluminescence lifetimes (the decay can be as long as 500 ns [88]). All of these factors make them preferential for a range of important applications. It is also important to note that the seeded growth method is the most employed as a means to synthesize Cu-based spherical core/shell heterostructures.

CIS and CIS-based QDs are the most studied systems in this category. Berends and Stam have carried out detailed studies on the shell coating process through the seeded growth strategy [144]. They checked low and high reaction temperatures, and various reactive and unreactive Zn- and S-precursors in their work. The researchers found that low reaction temperatures favour etching, cation exchange, and alloying. Reactive S-precursors combined with unreactive Zn-precursor resulted primarily in etching, while the use of reactive S- and Zn-precursors resulted in a combination of etching and ZnS deposition, followed by alloying. High reaction temperatures and less reactive precursors favour cation exchange followed by alloying. Michalska et al. have explored the effects of varying amine ligands that were used in the reaction solution on the optical properties and morphology of the CIS/ZnS QDs [145]. Their works paved the way toward the design of improved synthesis methods of CIS/ZnS core/shell heterostructures. The main reports on the synthesis of CIS/ZnS core/shell structures are summarised in the Table 2 below.

The CISe/ZnS core/shell structure was prepared by Li and Pan for applications in QD sensitized solar cells with CISe_0.8_/ZnS_0.2_ and CISe_0.7_/ZnS_0.3_ NCs being prepared by using different amounts of Zn(OAc)_2_ and sulfur powder in the shell coating step. A large-scale synthesis of water-soluble CISe/ZnS core/shell QDs have also been demonstrated by Kang and Yang [155], giving a photoluminescence quantum yield (PLQY ) of obtained samples as high as 23.3%, using a straightforward preparation method. Recently, near-infrared emitting CISeS/ZnS colloidal NCs were reported by F.L. Lox and Dang [156]. Firstly, Cu_2−x_Se NCs were prepared through hot-injection, which was then followed by cation exchange to produce CISe NCs. Subsequently, a seeded-growth method was employed to coat a ZnS shell. Interestingly, it was found that in the shell coating step, a partial cation and anion exchange took place, producing the novel CISeS/ZnS core/shell structure. In another approach, ZnSe_1−x_S_x_ shell was coated onto CISe cores through a similar mechanism by Moser and Yarema [157]. It was found that Zn cations could only diffuse into the outermost atomic monolayer of the CISe core at reaction temperatures below 100 °C, although when the temperature was raised above 100 °C, the second monolayer also became thermally accessible and therefore could be filled with Zn cations.

**Figure 8:**
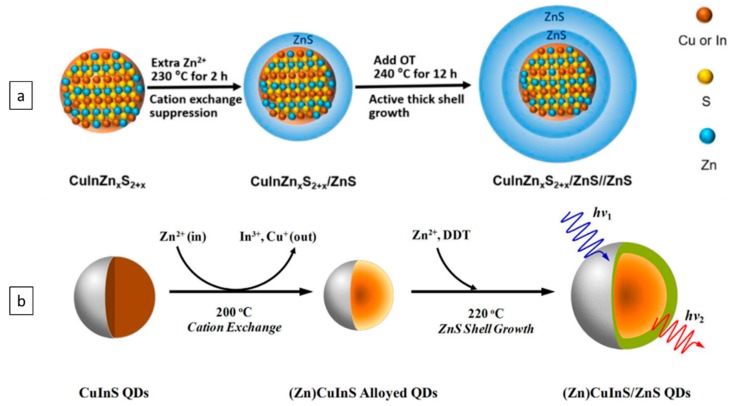
Scheme of (**a**) CIZS/ZnS/ZnS heterostructure. Reproduced with permission from [159]. Copyright Elsevier, 2018. (**b**) CIZS/ZnS heterostructure. Reproduced with permission from [74]. Copyright Nature publishing group, 2018.

For Cu-based quaternary core/shell structures, the techniques to synthesize CIZS/ZnS core/shell heterostructures have been much more developed than for the synthesis of corresponding CIZSe and CZTS because of its advantageous stability and optical properties. High-quality CIZS QDs can be achieved with advanced synthesis techniques, enabling the preparation of non-blinking (Zn)CIS/ZnS QDs with a high PLQY of 74% (Figure 8). This was done by Zhang and Dong through an in situ interfacial alloying approach [74]. CIZS/ZnS spherical heterostructure with tuneable emission can also be prepared using the seeded growth method by tuning the ratio between Cu/(Zn + In) [158]. Highly stable CIZS/ZnS/ZnS core/shell NCs with thick shells were also prepared by Wu and Wang (Figure 8) [159]. The presence of a blue shift commonly occurs during the ZnS shell-coating process, and this effect is either due to the etching of the core or the incorporation of Zn ions into the core. Interestingly, Guo et al. has developed an approach to address this unexpected blue-shift issue during the reaction for CIZS/ZnS core/shell structures [160], eliminating the majority of this effect.

### 5.2. Synthesis of Cu-Based Multicomponent Dot-in-Rod Heterostructures

This structure is based upon the incorporation of a core QDs in a shell of another semiconductor of a rod shape, with the position of the rod in this rod variable. The most well-known structure of this type is that of the CdSe/CdS dot-in-rod heterostructures with the growth mechanism of this synthesis, the optical properties and applications of this specific NR structure have been studied intensively. However, in contrast, there are only a limited number of reports concerning the synthesis of Cu-based multicomponent dot-in-rod structure, therefore, it is of great importance to understand the growth mechanism of this dot-in-rod structure to obtain Cu-based counterparts.

**Figure 9:**
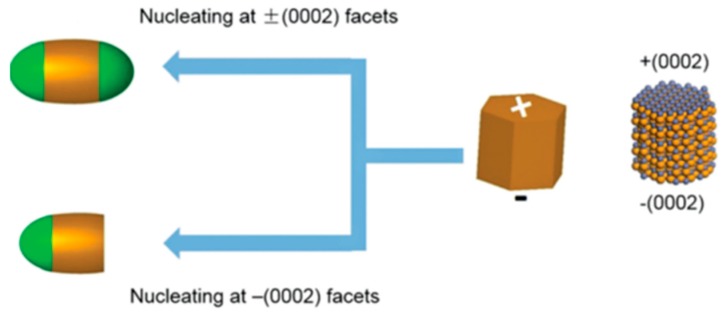
Scheme of the dot-in-rod heterostructure growth with different morphology. Reproduced with permission from [139]. Copyright RSC, 2014.

Generally, seeds with the hexagonal WZ crystal structure need to be firstly prepared for the preparation of a dot-in-rod nanostructure [161]. As for WZ seeds, there are only two opposite {002} facets that allow for the transition to epitaxial growth of zinc blend tips. The shell material can nucleate either on ± {002} facets or only on − {002} facets depending on certain reaction conditions [137]. Another point that is worth noticing is that the reactivity of the + {002} facet differs from that of the − {002} facet. Therefore, this gives access to localize the zinc blend tips selectively on one or both WZ ends or unequally localize on both ends as illustrated in Figure 9.

**Figure 10:**
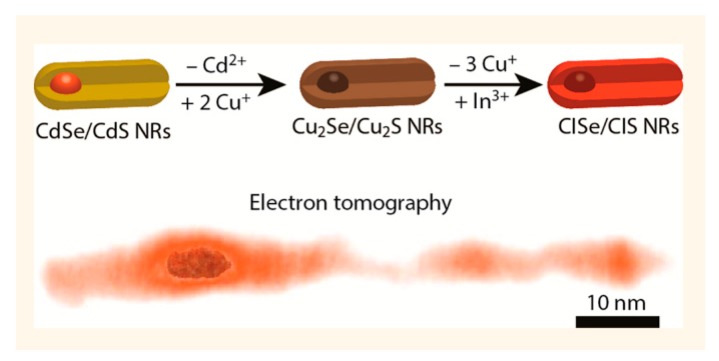
Scheme of the synthesis of the CISe/CIS dot-in-rod heterostructure. Reproduced with permission from [162]. Copyright American Chemical Society, 2015.

To date, there are only limited reports about the synthesis of Cu-based dot-in-rod heterostructures. For example, Stam et al. reported the first synthesis of Cu-based multicomponent dot-in-rod heterostructure [162] producing a CISe/CIS dot-in-rod nanostructure. However, the reaction process was quite complicated and involved Cd-based precursors (Figure 10) since the reaction proceeded via an initial CdSe/CdS dot-in-rod, which was prepared first. Following this, a Cu_2_Se/Cu_2_S dot-in-rod was obtained through complete cation exchange in which the resulting Cd was completely replaced by copper. At the final step, luminescent CISe/CIS was obtained by a partial cation exchange with indium incorporated into the rod structure. ZnSe/ZnS dot-in-rod also has been achieved through similar procedure, with complete cation exchange taking place to convert from CdSe/CdS to Cu_2_Se/Cu_2_S NRs, which was then followed by complete cation exchange to produce the ZnSe/ZnS dot-in-rod structure [132].

**Figure 11:**
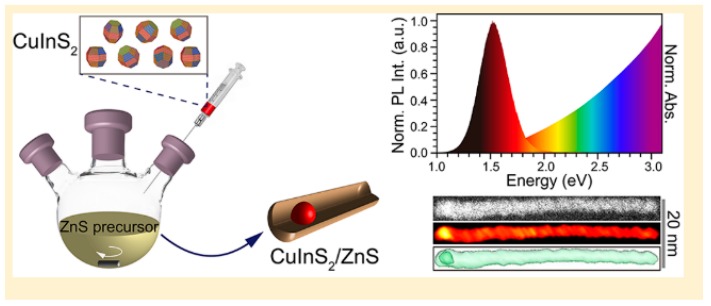
Scheme of the synthesis of the CIS/ZnS dot-in-rod heterostructure. Reproduced with permission from [163]. Copyright American Chemical Society, 2018.

Xia and Winckelmans were the first who have produced a CIS/ZnS dot-in-rod heterostructure via a seeded growth method [163]. WZ CIS seeds were synthesised first, that was followed by growth of ZnS tips (Figure 11). In this work, the growth mechanism for the particular Cu-based multicomponent heterostructure is similar to the growth mechanism of Cd-based dot-in-rod structures, with HDA used as a coordinating ligand. The obtained colloidal WZ CIS/ZnS dot core/rod shell heterostructures showed PL in the near infrared region with PLQYs of 20%, with emission being assigned to the CIS core QD. 

### 5.3. Synthesis of Cu-Based Multicomponent TP Heterostructures

There are a number of reports of Cd-based nano-TP structures [164]. Unfortunately, as with other Cd based structures, the presence of highly toxic heavy metals greatly impedes the practical application of these nanomaterials, so obtaining its Cu-based counterparts is imperative.

**Figure 12:**
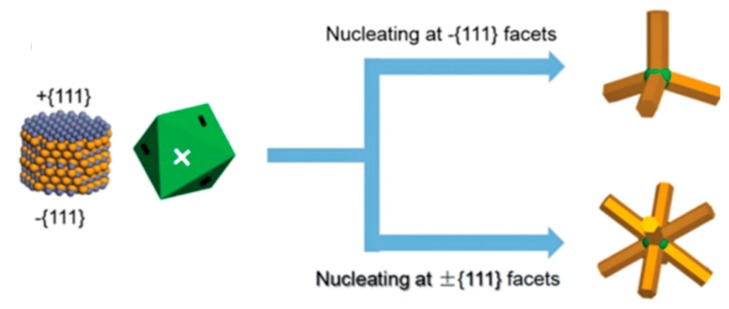
Schematic illustration of the growth of TP heterostructures. Reproduced with permission from [139]. Copyright Copyright RSC, 2014.

For this structure, a ZB core is necessary for the synthesis of these TPs. As shown in Figure 12, the ZB core has eight {111} crystalline facets that each can serve as nucleation points for the growth of WZ arms. When all the eight facets act as nucleation spots, octopods can be obtained [165,166], while when only four more reactive facets act as nucleation spots, this allows for epitaxial growth of WZ arms, enabling the growth of TPs [167,168,169].

**Figure 13:**
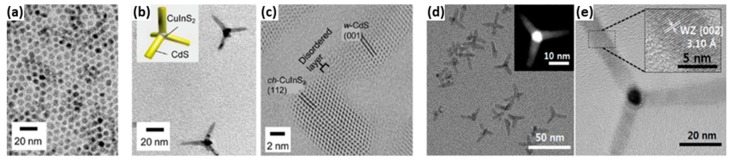
TEM images of CIS/ZnS TP heterostructures prepared with (**a**–**c**) CH CIS seeds. Reproduced with permission from [170]. Copyright RSC, 2016 (**d**–**e**) ZB CIS seeds. Reproduced with permission from [171]. Copyright American Chemical Society, 2018.

The techniques to prepare Cd-based TPs are very well documented [7]. As for Cu-based multicomponent counterparts, the Sakamoto group was the first one which reported incorporating a Cu-based core (CIS) into a TP heterostructure (Figure 13) [170] by initially forming the CIS seeds, and then the CIS/CdS TPs via a seeded growth method. They found that this TP structure possessed a quasi-type II band alignment, giving them a longer-lived charge. A particularly interesting point of this work is that the CIS seeds possessed a CH crystal structure, which is not consistent with the proposed growth mechanism above and in fact remains an issue to be explained.

Two years later, the Kim group was able to produce CIS/CdS TPs [171]. However, in contrast, in this case, the more traditional ZB CIS seeds were employed to obtain the final CIS/CdS TP heterostructures, producing significant PLQYs of up to 40%. Unfortunately, at present, absolute Cd-free Cu-based multicomponent TP heterostructures have not been achieved yet, though in theory, this structure should be readily accessible since the WZ structure of Cu-based cores, such as CIS and CIZS, can be readily grown as shells.

## 6. Applications of Cu-Based Ternary or Quaternary Quantum Nanostructures

QDs and particularly Cu based QDs have been tested in a range of important applications. Here, we focus on three areas below to best illustrate the particular importance of luminescent Cu based QDs, and to highlight clearly why particular anisotropic heterostructures show so much promise for future applications.

### 6.1. Luminescent Solar Concentrators

**Figure 14:**
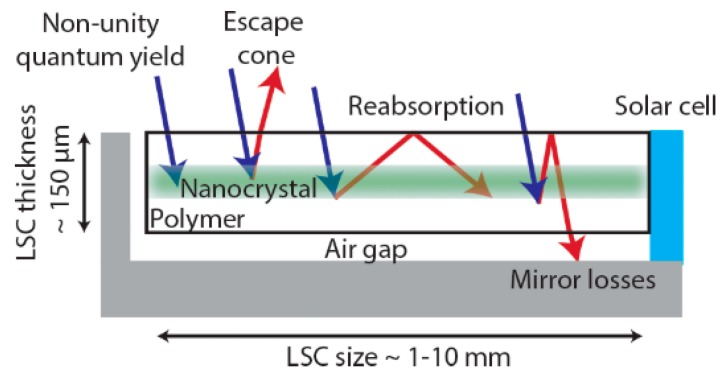
Scheme showing the energy loss pathways in a luminescent solar concentrator. Reproduced with permission from [172]. Copyright American Chemical Society, 2016.

Great concerns about environmental sustainability and global warming have stimulated intensive worldwide efforts, which have focused on the use of green renewable energy sources, such as wind and solar power. Hence, photovoltaics has received increased attention in the past decades, however, it is still a challenge to achieve high efficiency with limited fabrication costs at present. One of the approaches to fulfil these demands involves the use of luminescent solar concentrators (LSCs). The major goal of the LSCs is to concentrate solar radiation over a large area waveguide onto a small area of attached solar cells to increase the efficiency of photovoltaics and lower the cost of solar energy. An LSC is comprised of a planar waveguide containing embedded fluorescent materials with photovoltaics attached to the waveguide edges. In addition, Bragg mirrors are also sometimes incorporated into LSCs to enable better lateral propagation. Fluorescent materials absorb a certain fraction of the solar flux and re-emit this energy as luminescence with a higher wavelength (lower energy), with the remitted radiation trapped due to total internal reflection. There are essentially three pathways for energy loss: The first one is that a portion of the light exits through top surface of LSCs, termed the escape cone and is dependent on the refractive index of the matrix (Figure 14). The second possibility is the reabsorption of the photons by the fluorescent materials. In addition, if the Bragg mirror is far from perfect, then there will be a third energy loss pathway, which is called mirror losses. Due to the larger lateral area of the LSC to the edge area for solar cells, the photon density can be increased considerably, which is also known as the concentrator effect.

QDs are very promising fluorescent materials for the application in LSCs because of a number of advantages over traditional dyes. QDs with a high quality can be achieved easily due to the fast development of the colloidal synthesis techniques, enabling QDs to possess high luminescence quantum yields (even above 90%), tuneable absorption and emission based on the size, composition, and morphology, and excellent photostability (resistance to photobleaching). In addition, Cu-based multicomponent NCs are a much better choice than Cd- or Pb-based QDs when considering LSC applications due to the inherent environmental issue of heavy metal use. All these factors make Cu-based QDs meet many of the criteria for ideal fluorescent materials for LSCs. A large Stokes’ shift is a typical characteristic of Cu-based multicomponent QDs, therefore, the undesired reabsorption because of the small Stokes’ shift [173], which causes energy loss, can be eliminated via the use of Cu-based multicomponent QDs. Also, via a specific optimal heterostructure design, Cu- based QDs with an even larger Stokes’ shift can be prepared using a core-shell structure. In addition, the application of doped Cu-based QDs also possessing a large Stock shift are ideal for LSC applications, with the dopants (Mn^2+^, Co^2+^, Ni^2+^) producing an intraband energy level [174].

For practical applications, the fluorescent material in an LSC should display great resistance to photobleaching, therefore, type I core/shell systems would be the optimal choice regarding this issue. Some core QDs might be sensitive to the environment due to the potential oxidation under solar irradiation, therefore, this can be improved by coating the final NC in an inorganic shell (ZnS), producing a core/shell structure. In this case, the shell of ZnS, shows excellent photostability and therefore protects the core from the external environment, greatly enhancing photostability.

Thus, due to the discussed above reasons, Cu-based multicomponent core/shell heterostructures would be the ideal choice for LSCs applications. In spite of all the advantages of Cu-based heterostructures, there are still few reports in the literature related to their application in this area, though some excellent examples have been reported. 

Bright CIS/CdS phosphors have been tested for LSCs applications by Knowles and Kilburn [175]. The colloidal CIS/CdS QDs they prepared possessed large solar absorption, high PLQYs, and only moderate reabsorption, which led to CIS/CdS QDs outperforming CdCuSe QDs. However, this sample still contained the toxic Cd metal. Meinardi et al. demonstrated that the use of Cu-based multicomponent heterostructures allowed them to overcome many limitations of both organic dyes and more traditional Cd- or Pb-based QDs, including incomplete coverage of the solar spectrum, toxicity, and strong colouring of the LSC [176] (an optical power conversion efficiency of up to 3.27%). Li and Chen synthesized CIS/ZnS heterostructures for LSCs [177]. In their work, the as-prepared heavy metal free CIS/ZnS QDs possessed advantages of high PLQYs of 81% and a large Stokes’ shift of more than 150 nm. The optical efficiency of CIS/ZnS QDs-LSC was as high as 26.5%, with the power conversion efficiency of the QD-LSC-PV device reaching more than three-fold than that of a pure PMMA-PV device. Nearly reabsorption-free CISe/ZnS QDs were synthesized by Wu and the performance of this heterostructure was checked in a tandem LSCs [178]. They found that the power conversion efficiency improvement versus single-layer devices can exceed 50% (with bandgap-matched PVs).

All the above results indicate that highly effective performance can be achieved with these environmental-friendly, low-cost, easy processable Cu-based multicomponent heterostructures in standard or even tandem LSCs.

The use of morphology also offers some unique advantages when designing an LSC using heterostructured nanocrystals. For example, it has been simulated that if CdSe-CdTe NRs are aligned in the LSC with their long axis perpendicular to the top surface in the LSC, they emit anisotropic luminescence parallel to the top surface, thus reducing the escape of emitted light from the LSC [179]. There are a range of techniques that have been developed for the alignment of NRs in solution and the corresponding matrix, such as the solvent effect [180,181], thermal annealing [182], controlled drying [183], and electric field [184]. As for Cu-based multicomponent polytypic or heterostructured rod and TP structures, they have excellent light absorption abilities due to their large absorption cross section and carrier-guiding directions. All these unique morphology-dependent optoelectrical properties make them even more promising in LSC applications, although they have not been applied to this application at present and therefore remain a unique opportunity to further leverage the advantages of QD based LSC designs.

### 6.2. Bioimaging

**Figure 15:**
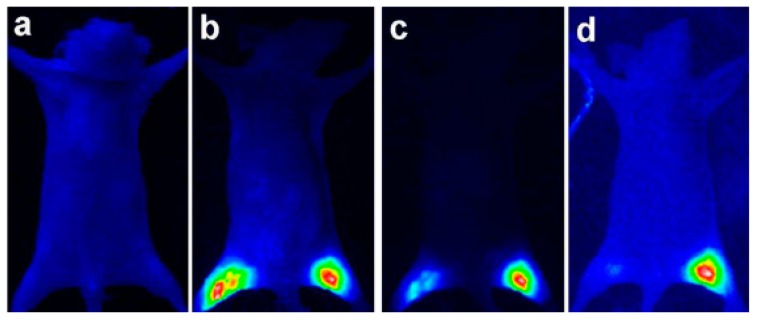
Multiplex near-infrared fluorescence imaging of mouse administered with two different NIR-emitting QDs-loaded micelles by subcutaneous injection (the right leg, 720 nm-emitting NCs; the left leg, 800 nm-emitting NCs): (**a**) Before injection, (**b**) $\lambda_{\mathrm{ex}}$ = 600 nm, a 700 nm long pass filter, (**c**) $\lambda_{\mathrm{ex}}$ = 660 nm, an 800 nm long pass filter, (**d**) $\lambda_{\mathrm{ex}}$ = 766 nm, an 800 nm long pass filter. Reproduced with permission from [195]. Copyright American Chemical Society, 2012.

Bioimaging refers to the visualization of biological tissues and processes (Figure 15). QDs have been studied extensively as the fluorescent probes because of their unique optical properties compared with traditional fluorescent dyes. Among them, Cu-based multicomponent NCs are more promising than the Cd-based counterparts due to the following reasons:

(1) Cu-based multicomponent QDs demonstrate less toxicity than Cd- or Pb-based QDs [185,186,187].
(2) The emission range of Cu-based multicomponent NCs are normally located at the near infrared region, which is more appropriate for many bioimaging applications [162,188]. In the visible spectra region, light scattering in biological tissues is distinct, which would restrict the spatial resolution and penetration depth of fluorescence imaging performance in that region.
(3) They possess relatively long PL lifetimes, which would help to improve the “signal to noise ratio” to obtain a better contrast [111].

To best take advantage of these properties of Cu-based QDs, it is also necessary for the fluorescent materials to have enhanced stability in the aqueous phase and against photobleaching. This can be done by coating with a ZnS shell onto the core to create a type I core/shell structure, with the aim to improve the effectiveness of the QDs. The evaluations of the chemical stability and cytotoxicity of CIS and CIS/ZnS have been done by Chen et al. [189]. They found that the intensity of photoluminescence has been enhanced by approximately 16 times after growing a ZnS shell around the surface of the CIS QD. In the cytotoxicity assay, the cell viability was more than 90% when the QD concentration ranged from 1 to 250 μg/mL, which was non-toxic to HeLa and OECM^−1^ cells. The X-ray absorption near-edge structure (XANES) results demonstrated the high chemical stability of CIS and CIS/ZnS QDs in Caenorhabditis elegans for a range of exposure times. Speranskaya et al. has studied the photostability and cytotoxicity of CIS/ZnS heterostructures [190]. It was found that the photostability of CIS/ZnS core-shell structures under constant UV illumination depends on the Cu/In ratio used for CIS core synthesis. Among them, CIS/ZnS with Cu/In equal to a 1:4 ratio, showed high photostability under UV illumination both in toluene and aqueous solutions. The as-prepared QDs were transferred to aqueous solutions by amphiphilic polymer encapsulation, with the CIS/ZnS QDs showing only slight cytotoxicity with all three polymeric shells. Confocal microscopy proved the penetration of CIS/ZnS NCs inside the cells, and indicated the low toxicity of the CIS/ZnS core/shell structures in general.

Highly luminescent, non-stoichiometric CIS/ZnS QDs have been synthesized for near infrared fluorescence bioimaging [191]. In this work, using in vitro and in vivo imaging sensitivity tests, the QDs were detectable to the depth of a few millimetres in an imaging phantom and in an intramuscular section of a mouse. This work demonstrated the feasibility of the bioimaging applications of the less-toxic, near infrared-emission CIS/ZnS system in deep biological tissues. Kim et al. prepared a CIS/ZnS core/shell structure [192] and the obtained QDs could be transferred into the aqueous phase. The authors claimed that the CIS/ZnS QDs were fabricated to an appropriate size for bioimaging (140 nm), which had potential applications in in vivo imaging. As for the quaternary core-based core/shell structure, Guo et al. carried out a one-pot synthesis of hydrophilic CIZS/ZnS QDs for vivo imaging [193] and the synthesis of CIZS/ZnS with inhibited blue-shift emission for tumor targeted bioimaging [160]. The as-prepared hydrophilic CIZS/ZnS QDs showed low cytotoxicity and offered a great opportunity for a direct in vivo imaging applications without phase transfer. As for the obtained CIZS/ZnS with inhibited blue-shift photoluminescence, these were transferred into the aqueous phase by a polymer coating technique and coupled with cyclic Arg-Gly-Asp peptide (cRGD) peptides. Following this, these QDs were injected via the tail vein into nude mice bearing U87 MG tumor. The results indicated that the signals detected in the tumor region were much more distinguishable when injected with CIZS/ZnS-cRGD rather than CIZS/ZnS QDs. All of these works demonstrated the advantages of Cu-based multicomponent core/shell structures for bioimaging applications.

Aside from luminesce, the application of multimodal particles is an emerging concept in bioimaging, and of great importance, enabling additional properties, such as a magnetic modality, in the same nanoprobe. The addition of paramagnetic functionalities, therefore, enables these nanoprobes to function as magnetic resonance imaging (MRI) contrast agents. Generally, there are three popular ways to combine QDs with magnetic resonance imaging (MRI) contrast agents, including combining them with magnetic nanoparticles, paramagnetic ion doping, or conjugating them with paramagnetic complexes, and have been previously demonstrated in Cd-based based QDs [194].

Recently, multimodal imaging has also been achieved in Cu-based multicomponent core/shell heterostructures. A method of Gd paramagnetic ion doping was explored by Yu et al. using CIS/ZnS NCs [196]. In their work, high quality bimodal bioimaging nanoprobes based on Gd -CIS/ZnS were prepared by a simple hot-injection method. The incorporation of Gd^3+^ into CIS/ZnS QDs led to a stable structure of Gd-CIS/ZnS, which produced enhanced MRI contrast, while still showing strong PL emission. Cheng et al. produced dual-modality Poly(maleic anhydride-alt-1-octadecene) (PMO) coated CIS/ZnS QDs by conjugating the PMO coated CIS/ZnS QDs with diethylenetriaminepentaacetic acid gadolinium(III) dihydrogen salt hydrate (Gd(III)-DTPA) [197]. A clear, positive, and increasing contrast enhancement of magnetic resonance signals was observed at increasing Gd (III) concentrations. This dual-modality nanoprobe also demonstrated excellent biocompatibility and negligible cytotoxicity. A bimodal contrast agent was successfully developed by chelation of the gadolinium ion to DTDTPA-modified CIS/ZnS QDs by Yang et al. [198]. It was proven that the as prepared CIS/ZnS QDs conjugating Gd(III) chelates had excellent capability to enhance the NIR fluorescence and MR imaging. These works are excellent examples of possible approaches to take full advantage of Cu-based multicomponent core/shell QDs for biological in vitro and in vivo studies. 

**Figure 16:**
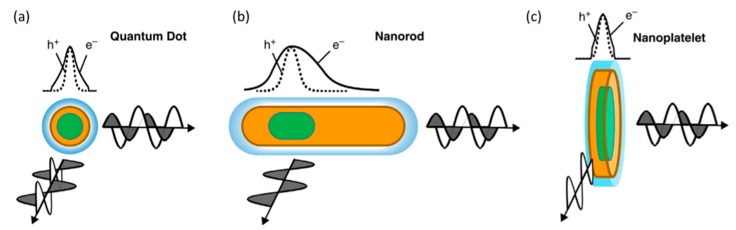
Illustrations of nanocrystal structures show wave functions of electrons and holes, and depictions of emission light polarization. Reproduced with permission from [199]. Copyright Elsevier, 2014.

NC heterostructures with rod, tetrapod, and plate morphologies, are greatly appealing to a range of bioimaging applications. For example, NRs demonstrate a unique interaction with polarized light due to the electron and hole being polarized along the long axis, producing linearly polarized light emission that can be used to detect particle orientation (Figure 16) and particle location via fluorescence polarization spectroscopy. NPLs are a family of QDs that are nearly planar and atomically flat, possessing narrow excitation and emission bands [199], making them an excellent candidate for multiplexed bioimaging applications, due to the enhanced capability to detect multiple signals. It has also been determined that rod like structures diffuse faster through porous media than dot shaped particles when measuring per weight and have a lower nonspecific cellular uptake, properties which are ideal for improving the imaging of cell plasma membranes [199]. This range of properties related to the anisotropic nature of NPs make them intriguing for a wide range of bioimaging uses.

### 6.3. Quantum Dot Based Lighting Emitting Diodes

Due to the wide tuneable emissive range and high quantum yields, quantum dots are excellent candidates for a range of architectures of lighting emitting diodes (LEDs), which either utilise QDs as down converters of a blue emitting conventional LED, termed a QDLED in the design, or through the use of QDs in an electroluminescence (EL) device design, creating an EL QD-LED.

In addition, as with other applications discussed previously, this field initially developed with the use of Cd based QDs, and then progressed towards the elimination of toxic metal use, and so the field has found huge success in recent times through the use of a range of Cu based ternary and quaternary QDs. Cu ternary and quaternary quantum dots are an excellent fit for a range of LED applications due to their tunability across the entire visible range, while achieving high quantum yields. A range of excellent reviews have covered this important field in the past number of years in great details [200,201,202].

Down converter LEDs are an area which has matured exceptionally, with Cu based QD devices showing excellent performance at present. In these designs, a blue emitting LED is used as the light source, upon which is placed a transparent matrix, typically a form of SiO_2_ resin, which is doped with variable concentrations of QDs. In this design, the blue LED emits, short wavelength photons, some or all of which are absorbed by the over-coated QD doped resin. Depending on the design, these QDs then reemit light upon excitation, giving the emission colour of the specific QDs utilised. For these designs, a range of Cu based QDs have been tested, including CuInS_2_/ZnS [151,152,203,204,205], CuGaS_2_ [206,207], and CuInZnS_2_/ZnS [208], along with CuSnInS [209] and Mn doped CuInZnS [210]. The performance of these devices heavily relies upon the PLQY of the utilised QDs, which ranges between 70%–80% in these QD based systems and whether there is a loss upon incorporation into the transparent matrix used, or if this PLQY is reduced overtime due to photobleaching effects. Another important characteristic is the question of reabsorption, which lowers the efficiency of the devices; therefore, some work has explored the use of dopants to increase the Stokes’ shift significantly, with Mn doped CuInZnS being an excellent fit for this [210]. 

In contrast to down converted QD LEDs, the field of electroluminescent QD LEDs remains still a field full of potential, with the designs showing significant progress, while many challenges remain to be tackled for commercialisation to take place. The devices take full advantage of the use of a semiconducting fluorescent nanoparticle, by using the inherent property of electroluminescence of fluorescent QDs. These devices are based upon the injection of charge carriers into the emissive layer, the QDs, at which point the charge carriers recombine, releasing the excess energy in the form of emitted light. This design allows for, in theory, much greater efficiency in comparison to down converter LEDs and allows for easier miniaturization of the QD-LED also. Unfortunately, disadvantages or at least complications come with this design also, since the device must be carefully designed so as charge carriers can be efficiently combined in the emissive layer, avoiding recombination via non-radiative routes, or loss of charge carriers while transporting them. This all means, as with solar cell designs, that multiple layers of different semiconductors must be chosen so as to drive this process to best take place at the highest efficiency, with the QD sandwiched between two charge transport layers at least. Currently, the most effective designs incorporate a layer to transport holes efficiently, typically a p-type organic semiconductor typically composed of poly(ethylenedioxythiophene): polystyrene sulfonate (PEDOT:PSS), and another layer to transport electrons efficiently, an n-type semiconductor metal oxide; typical examples would be ZnO, with the QD layer sandwiched in-between these.

A range of Cu based QDs have been tested in these designs, including Mn doped CuInZnS [211], CuInS_2_/ZnS [79,212,213,214], CuInSe_2_/ZnS [28], CuInZnS/ZnS [215], and CuInGaS/ZnS [216]. In addition, mixed systems exist using CuInS_2_/ZnS and ZnCdSe/ZnS QDs [217] or CdZnS/ZnS [218] have also been reported. A common trend in these systems is the use of synthesis, which optimise for high PLQY systems, with the dominant approach synthetically being to produce a type band alignment using ZnS on the core, therefore, increasing the PLQY and the resistance to photobleaching. In addition, it was shown that the control of [Cu]/[In] molar ratio in CuInS based nanocrystals can be effectively applied to achieve the improved electroluminescence performance [79].

**Figure 17:**
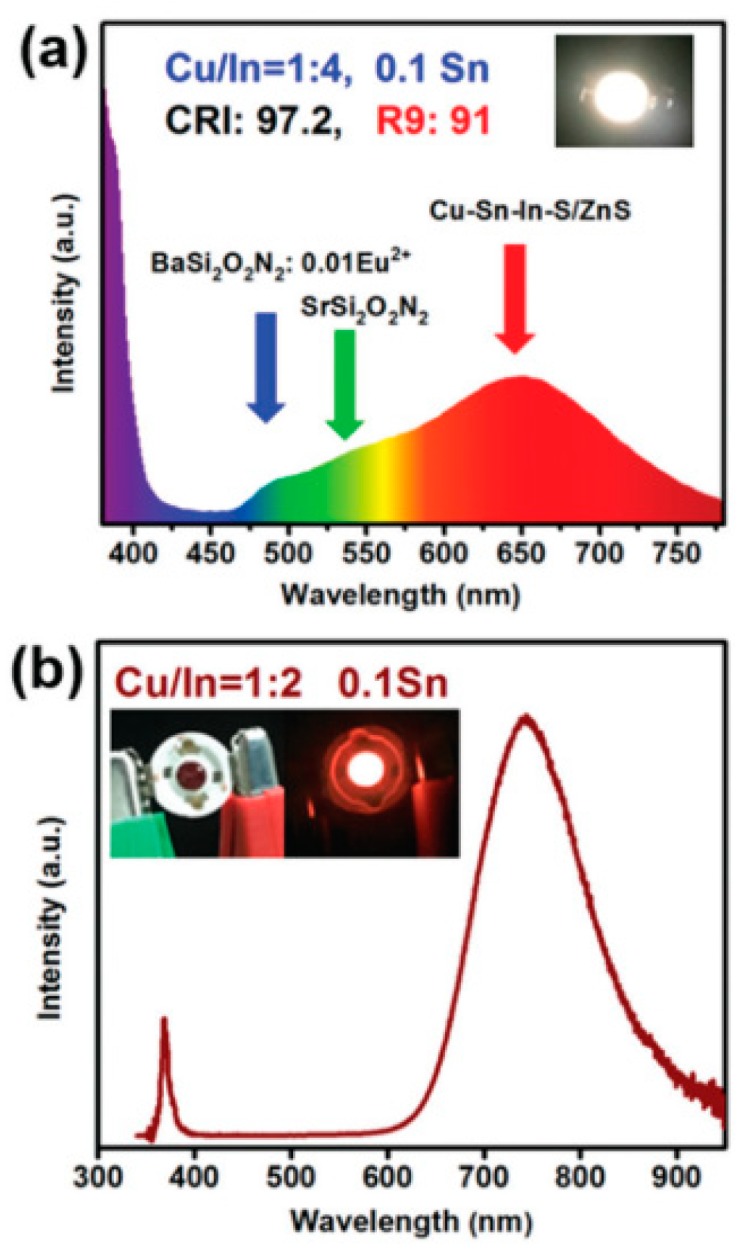
Electroluminescence (EL) spectra of (**a**) white light emitting diode (wLED) and (**b**) NIR LED devices combined with CTIS/ZnS QDs (0.1 mmol Sn) with the Cu/In molor ratios of 1/4 and 1/2, respectively. Reproduced with permission from [209]. Copyright RSC, 2018.

White light LEDs are an important class of LEDs aiming to produce emission as close as possible to blackbody emission at ≈ 6000 K, and therefore simulate sunlight to act as a replacement for a range of lighting sources used at present, including Cu–Sn-In–S based QDs [209] (Figure 17). A number of various Cu based QDs have also been demonstrated as promising white light sources [151,152,203,204,205,206,208,210,211,217,219,220,221,222], either via the use of down converting [152,203,204,205,206,207,208,209,210,219,220,221] or electroluminescence [211,217,218] s. These devices are achieved by either combining a number of luminescent sources or via the use of a single broad emission sources. Cu based QDs are an excellent fit for this type of light source due to the inherent broadness of the QD emission relative to other QDs. In addition, upon inclusion of dopants into the structure, the emission can be even further broadened, using Mn [210,211], Sn [209], or Ga [206], for example. In addition, the alternative approach of multiple luminescent dyes, can be easily achieved via either varying the stoichiometry of the QDs or via the size of the QDs. 

The application of nano-heterostructures is widely presented in the field of QDLED with the use of core/shell structures of type I band alignments showing significant success. In contrast, the effects of varying heterostructure morphology is only now gaining attention as a novel approach to further improve upon theses exciting devices. As with many areas of QD applications, Cd-based QDs have led the way in this investigation of anisotropic structures, with a number of studies showing some exciting results through the use of dot in rod structures of CdSe/CdS [223,224], CdSe NPLs [225,226,227,228], and CdSe/CdS TPs [229] in a number of LED designs. The unique advantages gained from the use of heterostructures have been very effectively covered in a recent review of QD LED designs [201], with polarization, alignment, and narrow emission being some of the highlights; some examples are detailed below to illustrate this point. CdSe/CdS dot in rods have been aligned on the surface of water to then enable the production of an emissive layer in a polarised electroluminescent device [223]. A CdSe_1−x_S_x_ NPL based LED has also been demonstrated showing the narrowest emission spectrum (FWHM of 12.5 nm) for any colloidal semiconductor LED [225]. Also, dual wavelength electroluminescent has been demonstrated using CdSe/CdS tetrapods, with two peaks associated with the core and arms of the heterostructure originating from distinct peaks, a property at present which seems unique to this specific morphology [229].

In addition to these core-shell anisotropic heterostructures, some very interesting results have recently been reported on the use of a relatively new nanomaterial, CdS/CdSe/ZnSe, termed as a double heterojunction nanorod for the design of some of the highest performing EL QDLEDs to date [230,231,232,233]. In this work, a CdS nanorod was initially grown, then CdSe was grown at two tips of the formed CdS nanorod, which was followed by the shell coating of ZnSe, forming two heterostructures at each end of the nanorods. Due to the morphology and the band alignments, these nanomaterials have demonstrated excellent performance.

## 7. Conclusions and Future Outlook

Thus, this review has considered recent developments in synthesis, optical studies, and applications of luminescent Cu-based chalcogenide multicomponent QDs. The complexity regarding the composition, morphology, and optical features in these materials was elucidated in detail, providing significant insights about the overall status and developing trends in this important area. 

The controlled hot-injection synthesis is still the most used method for the preparation of various Cu based QD nanostructures. This process normally involves the initial formation of binary Cu_2_S or Cu_2_Se precursor particles, followed by their transformations into ternary or quaternary nanostructures. In addition, the ion-exchange approach is a very promising technique for the preparation of anisotropic (e.g. platelet-like) nanostructures. We also believe that solvothermal methods could potentially be used for the synthesis of anisotropic Cu based QD nanomaterials, but a very significant future work is necessary in order to develop these techniques for controlled and reproducible preparation of complex ternary or quaternary nanostructures.

Concerning the underlying PL emission mechanism of Cu based QDs, there seems still to be no complete consensus reached in the literature, with additional work needed to be carried out to fully elucidate the important underlying processes. Once this problem is entirely resolved, a greater opportunity to further control the optical behaviour of these QDs and therefore tackle the corresponding photo-blinking and bleaching issues, which are significant roadblocks for important applications, such as QD-LEDs (QLEDs), lasers, solar concentrators, single-photon sources, and so forth. The composition and morphology also both have a huge influence on the optical properties of Cu-based multicomponent QDs, hence it is of great importance to control the versatility of both and to fully relate the complex interplay, again via more detailed studies of the huge range of structures already produced. In addition, a rich direction for novel science exists in study to fully map the possible and viable synthetic routes to make Cu-based multicomponent anisotropic heterostructures more available.

Regarding applications, Cu-based multicomponent polytypic or heterostructured rod and TP structures have excellent light absorption abilities due to their large absorption cross- sections and carrier-guiding directions, all of which make the unique morphology-dependent optoelectronic properties even more promising in LSC applications. Therefore, further development in the synthesis of these Cu-based nanostructures will be necessary to achieve significant increases in QD LSCs performance. In contrast, the bioimaging applications of these anisotropic NCs have not yet been explored extensively even for the much studied Cd-based counterparts, with few reports in the literature. An intriguing example is the application of CdSe/CdS/ZnS double shell NRs, which were exploited as biolabeling probes by Deka et al. It was found that the bright CdSe/CdS/ZnS double shell NRs (PLQY = 75%) were brighter and less toxic than the starting CdSe/CdS NRs in cell imaging [234]. However, all Cd containing QDs are potentially highly toxic and, therefore, their replacement with similar Cu-based Cd-free nanostructures will be very important for further development of any biomedical applications. Despite great potential, this area is currently under developed, though we are certain that a number of important opportunities lie in the fields of bioimaging, biosensing, and medical diagnostics using anisotropic non-toxic Cu based NCs. Also, anisotropic Cu based semiconductors at present have not been applied in the field of QD based LEDs either, but judging on the exciting results presented by cadmium-based systems, an outstanding opportunity exists in this field also, especially if an analogous double heterojunction Cu based nanorod could be developed and tested. Overall, an enormous amount of work still lies ahead to fully realize these very promising nanomaterials, but also based on the results to date, a range of exciting opportunities exists for ambitious researchers too. 

## Figures and Tables

**Table 1:** Properties of selected ternary and quaternary metal chalcogenide materials (at 300 K). Reproduced with permission from [24]. Copyright American Chemical Society, 2016.

Compound	Band Gap (eV)	Crystal Structure	Space Group	Lattice Parameters
CIS	1.53	CH	I-42d	a = 5.22 c = 11.12
CISe	1.05	CH	I-42d	a = 5.61 c = 11.02
CZTS	1.50	KS	I-4	a = 5.45 c = 10.86
CZTSe	1.02	KS	I-4	a = 5.61 c = 11.20

**Table 2:** Overview of the synthesis of CIS/ZnS heterostructures.

Precursors for Core	Ligands and Solvent for Core	Precursors for Shell	Ligands and Solvents for Shell	Dia of Core/Shell Structure (nm)	PLQY (%)	Ref.
CuI, In(Ac)_3_, DDT	DDT, ODE	ZnEX, Zn(St)_2_	ODE, DMF, toluene	2.8	50	[92]
CuI, In(Ac)_3_, DDT	DDT, ODE	Zn(Ac)_2_, DDT	TOA, OA	4–5	61.4	[11]
CuCl_2_, InCl_3_, DDT	DDT, ODE	ZnCl_2_, DDT	DDT	6–10	43	[146]
CuI, In(Ac)_3_, DDT	DDT, ODE	Zn(Ac)_2_, DDT	DDT	4.65–5.7	74	[147]
CuI, In(Ac)_3_, DDT	MA, ODE	Zn(Ac)_2_,	MA, ODE	4.2	65	[148]
CuI, In(Ac)_3_, DDT	DDT, ODE	Zn(St)_2_, ZnEX,	ODE, DMF, toluene, H_2_O	2.4–3.6	65	[98]
CuI, In(Ac)_3_, DDT	DDT	Zn(CH_3_COO)_2_ · 2H_2_O, DDT	DDT, OA, ODE	3.0–3.4	86	[149]
CuAc, In(Ac)_3_, DDT,	DDT, ODE	Zn(Ac)_2_, DDT	DDT, ODE	4.3	80	[150]
CuI, In(Ac)_3_, DDT	DDT	Zn(St)_2_, DDT	OA, ODE	5–10	60	[151]
CuI, In(Ac)_3_, DDT	DDT	Zn(St)_2_, DDT,	DDT, ODE	3.2–4.0	68–78	[152]
CuI, In(Ac)_3_, DDT	DDT, ODE	Zn(St)_2_, ZnEX	ODE, DMF, toluene	7	60	[153]
In(St)_3_, CuAc, S	OA, DDT, ODE	Zn(Ac)_2_, Zn(St)_2_, DDT	OAm, ODE, OA	12.5	50 ± 5	[154]

Abbreviations: Dodecanethiol (DDT), Octadecene (ODE), Trioctylphosphine (TOP), Oleic acid (OA), Glutathione (GSH), 3-mercaptopropionic acid (MPA), Zinc ethylxanthate (ZnEX), Trioctylamine (TOA), Dimethylformamide (DMF), Myristic acid (MA), and Oleylamine (OAm).

## Abbreviations

ASE	Amplified spontaneous emission
CGeS	Cu-Ge-S
CGS	Cu-Ga-S
CIGS	CuIn_1−x_Ga_x_S
CH	Chalcopyrite
CIS	Cu-In-S
CISe	Cu-In-Se
CIZS	Cu-In-Zn-S
CIZSe	Cu-In-Zn-Se
CTS	Cu-Sn-S
CTSe	Cu-Sn-Se
CZTS	Cu_2_ZnSnS_4_
CZTSe	Cu_2_ZnSnSe_4_
DAP	Donor-acceptor pair
DDT	Dodecanethiol
DMF	Dimethylformamide
EL	Electroluminescence
FWHM	Full width half max
GSH	Glutathione
HDA	Hexadecyl amine
HWHM	Half width half max
KS	Kesterite
LED	Light emitting diode
LSC	Luminescent solar concentrator
MA	Myristic acid
MPA	Mercaptopropionic acid
MRI	Magnetic resonance imaging
NC	Nanocrystal
NIR	Near-infra red
NPL	Nanoplatelet
NR	Nanorod
OA	Oleic acid
OAm	Oleylamine
ODE	Octadecene
PL	Photoluminescence
PLQY	Photoluminescence quantum yield
QD	Quantum dot
ST	Stannite
TOA	Trioctylamine
TOP	Trioctylphophine
TOPO	Trioctylphosphine oxide
TP	Tetrapod
WZ	Wurtzite
XANES	X-ray absorption near-edge structure
ZB	Zinc blende
ZnEX	Zinc ethylxanthate
